# The effects of 16-weeks of prebiotic supplementation and aerobic exercise training on inflammatory markers, oxidative stress, uremic toxins, and the microbiota in pre-dialysis kidney patients: a randomized controlled trial-protocol paper

**DOI:** 10.1186/s12882-020-02177-x

**Published:** 2020-11-26

**Authors:** Samuel A. Headley, Donna J. Chapman, Michael J. Germain, Elizabeth E. Evans, Jasmin Hutchinson, Karen L. Madsen, Talat Alp Ikizler, Emily M. Miele, Kristyn Kirton, Elizabeth O’Neill, Allen Cornelius, Brian Martin, Bradley Nindl, Nosratola D. Vaziri

**Affiliations:** 1 Exercise Science & Athletic Training Department at Springfield College, 263 Alden Street, Springfield, MA 01109 USA; 2 Renal and Transplant Associates of New England, Springfield, USA; 3 Department of Gastroenterology, University of Alberta, Edmonton, Canada; 4 Vanderbilt University Medical Center, Nashville, USA; 5 School of Psychology, Colorado Springs, Colorado USA; 6 Neuromuscular Research Laboratory/Warrior Human Performance Research Center, Department of Sports Medicine and Nutrition University of Pittsburgh, Pittsburgh, USA; 7 University of California Irvine, Irvine, USA

**Keywords:** Resistant starch, Inflammatory markers, Oxidative stress, Uremic toxins

## Abstract

**Background:**

Chronic kidney disease (CKD) is characterized by dysbiosis, elevated levels of uremic toxins, systemic inflammation, and increased markers of oxidative stress. These factors lead to an increased risk of cardiovascular disease (CVD) which is common among CKD patients. Supplementation with high amylose maize resistant starch type 2 (RS-2) can change the composition of the gut microbiota, and reduce markers of inflammation and oxidative stress in patients with end-stage renal disease. However, the impact of RS-2 supplementation has not been extensively studied in CKD patients not on dialysis. Aerobic exercise training lowers certain markers of inflammation in CKD patients. Whether combining aerobic training along with RS-2 supplementation has an additive effect on the aforementioned biomarkers in predialysis CKD patients has not been previously investigated.

**Methods:**

The study is being conducted as a 16-week, double-blind, placebo controlled, parallel arm, randomized controlled trial. Sixty stage 3–4 CKD patients (ages of 30–75 years) are being randomized to one of four groups: RS-2 & usual care, RS-2 & aerobic exercise, placebo (cornstarch) & usual care and placebo & exercise. Patients attend four testing sessions: Two baseline (BL) sessions with follow up visits 8 (wk8) and 16 weeks (wk16) later. Fasting blood samples, resting brachial and central blood pressures, and arterial stiffness are collected at BL, wk8 and wk16. A stool sample is collected for analysis of microbial composition and peak oxygen uptake is assessed at BL and wk16. Blood samples will be assayed for p-cresyl sulphate and indoxyl sulphate, c-reactive protein, tumor necrosis factor α, interleukin 6, interleukin 10, monocyte chemoattractant protein 1, malondialdehyde, 8-isoprostanes F2a, endothelin-1 and nitrate/nitrite. Following BL, subjects are randomized to their group. Individuals randomized to conditions involving exercise will attend three supervised moderate intensity (55–65% peak oxygen uptake) aerobic training sessions (treadmills, bikes or elliptical machine) per week for 16 weeks.

**Discussion:**

This study has the potential to yield information about the effect of RS-2 supplementation on key biomarkers believed to impact upon the development of CVD in patients with CKD. We are examining whether there is an additive effect of exercise training and RS-2 supplementation on these key variables.

**Trial registration:**

Clinicaltrials.gov

**Trial registration#** NCT03689569.

9/28/2018, retrospectively registered.

## Background

Cardiovascular disease (CVD) is the major cause of death among patients with chronic kidney disease (CKD) [1]. This increased risk of CVD is linked to the accumulation of uremic toxins, derived from abnormal bacterial growth within the gut (Rossi, Johnson, Morrison, Pascoe, & Jeff, 2018). Furthermore, CKD is marked by systemic inflammation and high levels of oxidative stress [2–4]. High levels of inflammation and oxidative stress are both thought to be key factors in the progression of CKD and in the development of CVD among patients with CKD [5, 6]. The uremia found in CKD patients is believed to damage the tight junctions between epithelial cells in the intestinal walls resulting in a more porous gut barrier, allowing toxins from the gut microbiota to leak into the systemic circulation of CKD patients. This would normally be prevented in healthy individuals by an impenetrable gut barrier. These enterotoxins trigger an immune response, which results in a heightened state of inflammation [5, 6].

CKD patients are typically prescribed diets to restrict the intake of potassium and phosphorus; this restriction leads to a reduction in the consumption of soluble fiber, which is known to have several health benefits [7–9]. There is evidence from both animal and human studies, primarily in persons with end-stage renal disease (ESRD), that supplementation with high amylose maize resistant starch type 2 (RS-2), a prebiotic, leads to a reduction in inflammation and oxidative stress [10–12]. Certain strains of beneficial bacteria utilize resistant starch as a nutrient to produce short chain fatty acids (acetate, propionate, and butyrate), which serve as nutrients for intestinal epithelial cells, promoting the repair of the altered tight junctions and aiding in the reduction of harmful bacteria [10, 12]. Consequently, fewer microbial components leak through the intestinal epithelium and enter the systemic circulation and the inflammatory response is reduced [10]. This effect has been demonstrated in a group of ESRD patients following an 8 week period of supplementation with RS-2 following which there was an increase in faecalibacterium, an anti-inflammatory gut microbe found at high levels in healthy individuals but reduced in a number of human diseases,and a reduction in both uremic toxins and markers of inflammation [11].

As demonstrated by Laffin et al. [11], supplementation with RS-2 leads to a reduction in uremic toxins in CKD patients. Key uremic toxins include indoxyl sulphate (IS) and p-cresyl sulphate (PCS) which are believed to accumulate systemically in CKD patients due to increased metabolism of some amino acids (tryptohan and tyrosine) and the kidney’s impaired ability to remove these substances [4, 13]. The accumulation of these uremic toxins also disrupts the balance between markers of inflammation and oxidative stress, which ultimately creates conditions that facilitate endothelial dysfunction and rapid atherosclerosis [4].

There is strong evidence that chronic levels of increased physical activity lead to reductions in systemic markers of inflammation: c-reactive protein (CRP), interleukin 6 (IL-6) and tumor necrosis factor alpha (TNF-α) that correlate with changes in gut microbes [14–16]. However, acute exercise leads to a marked transient increase in IL-6 concentrations which is believed to have an anti-inflammatory effect by increasing the anti-inflammatory cytokines IL-1 receptor antagonist (IL-1RA) and interleukin 10 (IL-10) and decreasing the pro-inflammatory cytokine TNFα [17]. IL-6 brings about these favorable changes in the inflammatory milieu via actions on monocytes and macrophages [16]. Chronic exercise training protects against low grade systemic inflammation implicated in the development of many non-communicable diseases [16]. Elevated levels of IL-6, at rest, have been associated with a greater risk of premature mortality in CKD patients [3]. Chronic moderate intensity aerobic exercise decreases resting levels of IL-6 while at the same time increasing IL-10 levels [3].

The impact of resistant starch, primarily RS-2, has been primarily demonstrated in hemodialysis patients [11]. There have been few, if any, reported studies of the impact of RS-2 in CKD patients not on dialysis on the aforementioned biomarkers. Other forms of prebiotics (e.g. fructans) have been investigated with varying effects [13]. Therefore, the primary aim of this study is the examine the impact of 16 weeks of supplementation with RS-2, in association with aerobic exercise training, in a sample of stage 3–4 CKD patients on markers of inflammation, oxidative stress, uremic toxins and on the composition of the gut microbiota. Our proposal is novel in that it attempts to address one of the root causes of the elevated inflammation in CKD patients (dysfunctional microbiome) and determine whether moderate aerobic exercise training, which has been shown to have anti-inflammatory effects in some studies with CKD patients, has an additive effect along with RS-2 supplementation.

We hypothesize that supplementation with RS-2 will lead to normalization of the composition of the microbiota in our sample of CKD patients and reduce key markers of inflammation, oxidative stress, and uremic toxins. Reductions in these biomarkers will be associated with favorable changes in cardiovascular variables (arterial stiffness, blood pressure and vasoactive substances). We also hypothesize that moderate intensity aerobic training will have an additive anti-inflammatory effect along with the consumption of the resistant starch. The study is being conducted as a double-blind, placebo controlled, parallel arm randomized clinical trial.

## Methods/design

### Study setting

The study is being conducted at an academic institution in association with a local nephrology practice. The study has been reviewed and approved by the Institutional Review Board of the academic institution. This Board assumes the responsibility for monitoring the study. Any adverse events are required to be reported to the IRB in compliance with policies of this Board. Any changes to the original protocol are reviewed and approved by the IRB before implementation in this study. If additional analyses are to be performed that are not listed in the original consent form, consent will be required prior to performing any new analyses.

### Eligibility criteria

#### Inclusion criteria

Sixty stage 3–4 CKD (GFR 15–59 ml/min/m^2^) patients between the ages 30–75 years are being recruited for this study, which is being completed over a 3-year period. Other inclusion criteria include the ability of the individual to comply with all aspects of the study protocol and to be able to give consent independently.

#### Exclusionary criteria

Patients are excluded if they have any of the absolute contraindications to exercise as defined by the American College of Sports Medicine (ACSM) [18]; if they have received a kidney transplant; if they have received antibiotic therapy within the previous month, or if they have taken prebiotic or probiotic supplements within the month prior to the start of the study [19]; if they have any gastrointestinal condition that prohibits their use of resistant starch [19], or if they have been involved in a structured exercise program (i.e., exercising 3 days per week at a moderate intensity for at least the previous 3 months). Individuals who are pregnant are also excluded from this study.

### Recruitment

A member of the research team (EE), has been given access to the patient database at the local nephrology practice participating in this study. She screens the list of patients and approaches those who meet the eligibility criteria for the study at their next scheduled appointment. While patients are waiting to see their nephrologist, she explains the details of the study to them. If they indicate a willingness to participate, she gives them the medical history form to take into the appointment with them. If the nephrologist approves of the patient’s participation in the study, EE obtains informed consent from the patient and enrolls them into the study at that time. Individuals are free to withdraw from the study at any time as stated in the IRB approved consent form. Individuals who experience adverse reactions from taking the supplement will be removed from the study without penalty.

### Confidentiality

All forms with participant information for this study are stored in a locked filing cabinet in the PI’s office with access limited to specified members of the research team. Upon entry to the study, participants are given a number and this number is then used on all documents, apart from the consent form and within the database. The study data are stored in a password protected database with access limited to the PI, the statistician and members of the research team tasked with data entry and verification.

#### Testing sessions and timeline

**Fig. 1:**
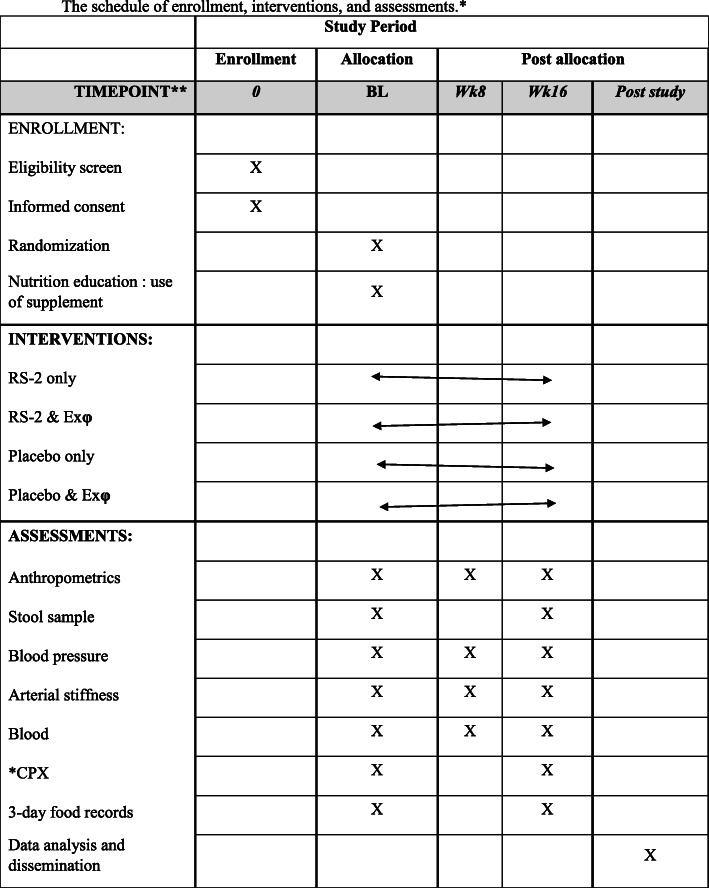
The schedule of enrollment, interventions, and assessments.*

Participants are asked to attend a total of four testing sessions (see Fig. 1), all conducted at the academic institution’s Human Performance Laboratory. During the first visit, anthropometric variables of, height (Detecto, Webb City, Missouri), weight and body composition via bioelectrical impedance (BC-418,Tanita,Tokyo, Japan) are assessed along with the determination of peak oxygen uptake (VO_2peak_) using a computerized metabolic cart (MAX-II, AEI Technologies, Pittsburg, PA), that is calibrated according to manufacturer’s specifications. The cardiopulmonary exercise test is performed on a motor driven treadmill with a Q-stress system (Welch Allyn, Skaneateles Falls, NY) using the Modified Bruce protocol. Resting cardiovascular (CV) variables (i.e., central and peripheral blood pressure, pulse wave velocity and pulse wave analysis) are assessed using applanation tonometry (SphygmoCor Excel, AtCor Medical Inc., Itasca, IL) after a 10-min rest period in the supine posture. One week later, patients return to the Human Performance Laboratory for their second baseline visit following at least an 8-h fast. On arrival, a blood sample is taken, and processed by spinning in a refrigerated centrifuge (4 °C, and spun at 3000 rpms for 15 min). The plasma is then removed and placed in 10 storage tubes and stored in a − 80° freezer for later analysis. Prior to visit 2, participants collect a stool sample at home and complete a 3-day food record documenting the brand, portion size, and preparation methods of all foods and beverages consumed. Both the stool sample and the food records are brought to the lab for visit 2. Following baseline testing, subjects are assigned randomly to one of 4 groups ensuring equal number of subjects in each group: Group A receives the prebiotic supplement with usual care but no prescribed exercise, Group B receives the prebiotic supplement and performs supervised aerobic exercise training at a moderate intensity for 16 weeks, Group C receives the placebo (cornstarch) supplement along with 16 weeks of moderate intensity aerobic exercise training, while group D receives the usual care and the placebo but no supervised exercise training. The randomization was performed by the study statistician prior to the start of the study.

#### Follow-up testing

All of the aforementioned variables (except the stool sample, and the VO_2peak_ test) are reassessed after 8 and 16 weeks of participation in the study. The final stool sample and the post-training VO_2peak_ test are done after week 16. All post-tests are performed at least 48- h following the completion of the final training session to avoid the residual effect of the final training session.

### Analysis of biomarkers

#### Analysis of inflammatory and oxidative stress markers

Venous blood samples are being collected at BL, Wk8 and Wk16. A 21- or 23-gauge needle (BD Vacutainer® Safety Lok and Vacutainer® one-use holder, Becton, Dickinson and Company, Franklin Lakes, NJ) is used to collect two, 10 mL of blood at each of the time point and placed into an appropriate collection tubes (EDTA Monoject Vacutainer, Coviden, Mansfield, MA) by trained personnel. Plasma is obtained from the EDTA tubes through centrifugation immediately after collection at 1500 g for 15 min at 4 °C. Plasma samples are being stored at − 80 °C until ELISA assays are conducted for the analyte panel, including: CRP, malondialdyhide (MDA), and nitrate/nitrite (Cayman Chemical, Ann Arbor, MI), Cytokine panel (TNFα, IL-6, IL-10), Enothelin-1, (Millipore, Burlington, MA), MCP-1 (Eagle Biosciences, Amherst, NH), and 8-isoprostane (Cell Biolabs, San Diego, CA). All samples will be measured in duplicate, in accordance with manufacturers’ recommended protocol and be within acceptable variance and above sensitivity provided from the manufacturer.

#### Analysis of uremic toxins

The concentration of IS and PCS in the plasma is measured by reverse-phase HPLC. In brief, 0.24 M sodium octanoate is added in plasma as a competitor to replace non-covalent binding of *p*-cresyl sulfate and indoxyl sulfate to albumin. After 5 min incubating at room temperature, the plasma is deproteinized by adding 4 parts of cold acetone to 1 part of plasma. The mixture is then centrifuged at 3000×*g* for 20 min at 4 °C and the supernatant is measured by HPLC (Waters 1525 binary HPLC system coupled with 2475 fluorescence detector) [20].

#### Analysis of stool samples

Stool will be used for microbial assessment by 16 s RNA sequencing (MiSeq platform, 2 × 300 bp). The pipelines of QIIME2 with DADA2 will be used to perform demultiplexing sequences, quality control, feature table construction and taxonomy assignment [21]. Taxonomic assignment (from kingdom to genus level) of the representative sequences of each sample will be performed using the Silva 132 pre-trained Naive Bayes classifier and the q2-feature-classifier plugin in the QIIME2 pipeline. Alpha-diversity will be calculated by observed species, Chao1, and Shannon indices using QIIME2. To compare microbial profiles between samples, weighted (quantitative) and unweighted (qualitative) variants of UniFrac and Bray-Curtis will be measured. Principal coordinate analysis (PCoA) will be applied on the resulting distance matrices to generate two-dimensional plots. Permutational multivariate analysis of variance (PERMANOVA) will be used to statistically test for significant differences of the microbial profile between groups. Hierarchical clustering analysis will be performed to show a visual interpretation heatmap of the similarity of microbial taxa and function gene among groups. In terms of downstream microbiome analysis, centered Log Ratio Transformation (CLR) will be applied to transform the microbiome composition. The longitudinal model (MetaLonDA) [22] and the generalized estimating equation (GEE) model [23] will be performed to compare the dynamics of microbial composition over time between groups. Within each arm, the repeated ANOVA with permutation test will be used to analyze microbial composition.

### Interventions

#### Nutrition protocol

Participants are randomized to receive a supplement of either resistant starch (intervention) or waxy cornstarch (control). The study dietitian (DC) is the only “unblinded” member of the research team, and is responsible for providing the correct supplement to participants, without revealing the randomization scheme to other team members or participants. At the completion of the study, DC reveals the contents of the supplement to the participant. The resistant starch is composed of 40% digestible starch and 60% resistant starch and is provided as Hi-Maize® 260 resistant starch (Hi-Maize 260, Ingredion, Bridgewater, NJ). The control is Amioca® corn starch (AMIOCA, Ingredion, Bridgewater, NJ), is nearly completely digestible. For week 1, the dose of supplements is 15 g/day. The dose is then increased to 33 g/day for weeks 2–16. Participants are instructed to blend their supplement into commonly consumed foods/beverages such as yogurt, soups, oatmeal, coffee, or juice. This daily dose was selected to provide a minimum of 30 g resistant starch/day as previously reported by Sirich et al. [9], and allow for the 10% variability in the sachet weight as determined by the commercial packing company. Sirich et al. [9] utilized a similar protocol with good gastrointestinal tolerance. Participants are provided with dosing suggestions and recipes to promote good compliance. Supplements are provided to participants as daily doses in sachets labeled as either “Supplement 1” or “Supplement 2”. Participants are issued half of their daily doses of supplement at visit 2, and the remaining daily doses of supplement are provided at visit 3. Participants are instructed to return any unused supplement at the completion of the study. A research assistant contacts the subjects biweekly during the study to determine if the subject had experienced any complications with consuming the supplement and to encourage compliance. Three-day food records are administered at baseline, and week 16 and entered into Food Processor software (ESHA) for nutrient analysis -to assess between-group differences in dietary intake of nutrients which may impact upon renal function (i.e. protein, potassium, phosphorus).

#### Exercise protocol

Individuals assigned to the exercise groups participate in supervised training (3 x per week at 55–65% VO_2_ peak for 45–50 min) for 16 weeks in the Wellness Center of the institution. Each exercise session begins with a 3–5 min warm-up at a low intensity and static flexibility exercises. Participants build up their exercise tolerance to complete 45–50 min of moderate intensity continuous aerobic exercise using a combination of exercise apparati, depending upon their preference. The heart rates that corresponded to 55–65% of measured VO_2_ peak from the exercise test are used to monitor exercise intensity along with perceived exertion to make sure that subjects are working within a safe, but effective range. At the completion of each workout, subjects are taken through a cool-down procedure involving static stretching. Blood pressure is assessed before and after all exercise sessions and if these values are abnormal for the individual, they are not allowed to exercise on that day. Blood glucose is assessed in persons with diabetes prior to and after each exercise training session. If the pre-exercise value is less than 100 mg/dl the individual is given a drink containing approximately 15 g of carbohydrate. However, if the baseline plasma glucose level exceeds 250–300 mg/dl with ketones present, the individual is not permitted to exercise on that day in compliance with ACSM guidelines [18].

### Outcomes

**The primary outcomes** of this study are systemic markers of inflammation**:** CRP,TNFα, IL-6, IL-10, MCP1, markers of oxidative stress, MDA, 8-isoprostanes F2a, uremic toxins, PCS and IS and the faecal microbiota composition. All biochemical markers will be assayed by well-established labs that are blinded to the group assignments of the participants.

**Secondary outcomes** include indices of vascular function; carotid-femoral arterial stiffness as pulse wave velocity (PWV)**,** resting peripheral and central SBP/DBP (measured by applanation tonometry), endothelin-1, and nitrate/nitrite.

### Statistical analyses

An Intention-To-Treat design will be used in this study. Effects of treatment on all dependent variables will be assessed by a 2 (prebiotic supplement or control) by 2 (aerobic exercise or control) by 3 (time) mixed factor ANOVA. The effect of the prebiotic supplement will be determined by examining the prebiotic supplement x time interaction, the effect of exercise will be determined by examining the aerobic exercise x time interaction, and the combined effect of the supplement and exercise will be determined by examining the 3-way supplement x exercise x time interaction. Significant interactions will be explored through simple effects analysis. The main variables of interest are inflammatory markers, particularly IL-6, and IL-10.

### Data auditing

Prior to performing the final study analyses, data will be checked by the statistician who will be assisted by research assistants in this process. These individuals will be blinded to the group assignment of the participants.

### Sample size estimates

A study investigating the effects of aerobic exercise on inflammation reduction [24] demonstrated effect sizes (Cohen’s *d*) varying between .40 and .77 for IL-6 and IL-10 and their ratio, which would be considered a medium to large effect. Few studies have investigated the effect of prebiotic supplements on inflammation, though similar magnitude effect sizes were reported for a prebiotic supplement intervention on changes in free circulating uremic solutes indoxyl sulfate and p-cresol sulfate, key indicators of adverse outcomes of CKD [9], though those changes were not found to be significant in that small sample study. Based on these studies, we anticipate medium effect sizes, and with power set at .80, and alpha set at .05, a sample size of 60 (15 per group) is predicted to be sufficient for detecting significant two-way interactions specified in the statistical analysis section. To account for any dropouts from the protocol, missing data will be addressed through multiple imputation, and results of all analyses averaged over multiple imputed datasets. All variables in the analysis will be examined for assumptions for all statistical tests. Data will also be examined in a per-protocol analysis, using only data from participants in all groups who completed 70% or more of their assigned exercise sessions and/or supplement use and who provided outcome data on the relevant variables.

### Dissemination policy

Upon the completion of data collection, the PI and the research team will endeavor to publish the findings of this trial in peer-reviewed journals whatever the outcome of the trial. The PI will also submit the findings to be presented at relevant professional meetings.

## Discussion

The purpose of the study is to examine the effect that 16 weeks of supplementation with the prebiotic, RS-2, has on the composition of the microbiota of a sample of stage 3–4 CKD patients. In addition, we will be monitoring the impact of this prebiotic on the levels of key uremic toxins (PCS & IS), markers of inflammation (CRP, TNF-a, IL6,IL10, MCP1), oxidative stress (MDA and 8-isoprostanes F2a,) and some cardiovascular parameters (arterial stiffness and blood pressure) that are known to impact upon the development of CVD. The potential additive effect of 16 weeks of moderate intensity aerobic exercise on the aforementioned variables will also be explored. Since many of the previous studies performed with RS-2 supplementation have been completed with ESRD patients, this study is one of the first to examine the impact of this supplement on the most prevalent stages of kidney disease, stage 3 & 4 [1].

The success of this protocol depends upon the adherence to two behavioral interventions, namely nutritional supplementation with RS-2 and aerobic exercise training for those randomized to the exercise groups. To enhance compliance to the nutritional aspects of the study, all participants have individual counseling sessions with the study dietitian to review various strategies to consume the product. At two-week intervals during the study, a research assistant is tasked with attempting to contact the participants to determine if there are any concerns with taking the supplement. If there are any such issues, the registered dietitian is informed and the issue is addressed with the participant as soon as it is feasible.

The inclusion of supervised exercise training is a strength of the study [25]. There is some evidence that exercise training alone has a favorable effect on the composition and function of the microbiota in some individuals [26, 27]. This information has not been previously examined in persons with CKD who are known to have a dysfunctional microbiota [28, 29]. Having participants directly supervised during each training session ensures that participants are given the guidance that they need to successfully complete their prescribed exercise training [25]. This also builds in a sense of accountability.

### Update

Due to the ongoing COVID-19 pandemic, we have been forced to make some changes to our original protocol. All of these changes have been reviewed and approved by the IRB of the College in addition to the department that oversees the grant. All recruitment is now being done remotely. Patients are being screened by the study recruiter (EE) who then gets permission from prospective participants for the PI to call to give details about the study. Once a participant agrees to enroll in the study they are sent the consent form and a COVID-19 waiver either electronically or in the mail. The participant must sign each of these prior to starting the study. The day prior to each research session, the PI calls the participant to complete a COVID-19 symptom screen which consists of a series of seven questions and confirm the details for the upcoming visit including the necessity of the participant to wear a mask. On the day of the visit, the PI, wearing full personal protective equipment, meets the participant outside of the building and takes a temperature reading before the participant enters the building. Following this, the PI continues to complete the study protocol as previously described.

The major change to the entire protocol is the removal of the exercise component. Participants no longer complete the graded exercise test nor are they assigned to any exercise intervention. This was necessary due to the shut down that occurred due to COVID 19 and due to the fact that individuals from outside of the College community are not allowed access to the training facilities where the exercise would have taken place. This was done in an effort to minimize the risk of participants or researchers contracting the COVID-19 virus.

Another adjustment that was made to the protocol was a switch to remote counseling by our dietitian. After visit 2, the participant no longer meets with the dietitian (DC) in person but rather receives dietary counseling over the phone regarding how to take the supplement.

All other aspects of the protocol remain in place. There are no plans to resume the exercise components of this particular study. Additional modification will be needed for the statistical analyses. Given the reduced number of participants completing the exercise portion of the protocol, the intended comparison between the prebiotic and exercise conditions will only be exploratory in nature. The effect of the prebiotic compared to placebo will be examined by a 2 (prebiotic vs. placebo) × 3 (time) mixed factor ANOVA.

## Supplementary Information

**Additional file 1.**

**Additional file 2.**

## Data Availability

The PI will have access to the completed data set which will be used for publications purposes only. It is therefore our intent to publish our data in reputable peer-reviewed journals so that the public can have access to our findings. We can make data available to those interested upon request.

## Abbreviations

CRP: C-reactive protein
CVD: Cardiovascular disease
CKD: Chronic kidney disease
ESRD: End-stage renal disease
RS-2: High amylose maize resistant starch type 2
IL-6: Interleukin 6
IL-10: Interleukin 10
MCP1: Monocyte chemoattractant protein 1
PWV: Pulse wave velocity
TNFα: Tumor necrosis factor alpha,
SBP: Systolic blood pressure
DBP: Diastolic blood pressure
