# Supplementary material for: The effects of 16-weeks of prebiotic supplementation and aerobic exercise training on inflammatory markers, oxidative stress, uremic toxins, and the microbiota in pre-dialysis kidney patients: a randomized controlled trial-protocol paper
Source: BMC Nephrol. 2020 Nov 26;21:517. doi: 10.1186/s12882-020-02177-x (PMC7689649; doi:10.1186/s12882-020-02177-x)
Supplement: Supplementary file 1 — Additional file 1. [file 12882_2020_2177_MOESM1_ESM.docx]

**CONSENT TO PARTICIPATE VOLUNTARILY**

**IN A RESEARCH INVESTIGATION**

**Exercise Science & Sport Studies Department**

**SPRINGFIELD COLLEGE**

**SPRINGFIELD, MA 01109**

**S. A. E. Headley, Ph.D.,**

**Investigator’s Name**

**_______________________ _________________**

**Subject’s Name Date**

**PROJECT TITLE: Effects of Prebiotic Supplementation on Inflammatory Markers, Vascular Function, Cognition, and Mental Well-being in Pre-Dialysis Kidney Patients**

You are being asked to participate in a research investigation as described in this form below. All such investigational projects carried out within this department are governed by the regulations of both the Federal Government and Springfield College. These regulations require that the investigator(s) obtain from you a signed agreement (consent) to participate in this project.

The investigator will explain to you in detail the purpose of the project, the procedures and/or drugs to be used, and the potential benefits and foreseeable risks of participation. You may ask the investigator any questions you may have to help you understand the project and you may expect to receive satisfactory answers to questions. A basic explanation of the project is written below.

If, after this discussion, you decide to agree to participate in the project, please sign this form on the line indicated below in the presence of a witness and the investigator or his designee.

1. **Purpose of Study**

The study described in this form has a number of purposes:

- The primary purpose is to determine the effect of consuming a cornstarch-based fiber supplement daily for 16 weeks along on markers of inflammation and oxidative stress.
- We will also be seeking to determine if the consumption of the fiber supplement, beneficially changes the functioning of your arteries.
- We will see if the fiber supplement has any impact upon the amount of time you spend being sedentary during the waking hours of the day.
- Another purpose of this study is to see if consuming the fiber supplement as described above, favorably changes physiological markers of stress in your body.
- We will see if the fiber supplement has any impact upon your mental health, cognitive function, and overall quality of life.

II. **The procedures to be used in this study**

Once you have obtained medical clearance and consented to participate in this study, you will be asked to attend **4 research sessions** at Springfield College.

**Visit 1**: During your first visit the following measurements will be taken on you.

- We will measure your height, weight, and body fat.
- We will ask you to complete a short computerized task to assess cognitive and emotional processing.
- After 10 minutes of supine rest, we will assess your blood pressure in both arms along with the stiffness of your vessels. In a following session, we will use the arm in which we found the higher reading.
- We will go over the details regarding the collection of your stool sample that you will need to bring with you to the next visit along with how to log the foods that you eat for a three-day period.
- We will also tape a monitor (using a non-allergic tape) to your right thigh and ask that you wear it for a seven-day period. This monitor will be waterproofed and can be worn while you shower. We only ask that you do not swim or bathe while wearing it. We will ask you to complete a diary that includes when you go to sleep and when you wake each day during the monitoring period.
- You will be paid $20 at the end of this visit.

**Visit 2:** At least **2 (7)** days following the first visit we will ask you to return to the lab.

- Prior to coming to the lab you will be asked to collect your stool sample following the procedures as described in your previous visit.
- You will also need to record all the foods that you eat and drink for 3 days and bring that information along with you to this visit.
- You will be expected to come to the lab after at least an **8 hour fast**. That means that you will not be allowed to eat any solid food but **we encourage you to drink water.** No coffee, teas, juices or colas are to be consumed and if possible, please hold off on taking your medications until after completing the testing session. We want you to repeat this process each time you have your blood drawn.
- After 10 minutes of rest lying on your back, we will assess your blood pressure in the arm in which we got the higher reading in visit 1, and the stiffness of your vessels.
- After the blood pressure measurements have been taken we will take a blood sample (approximately 3 teaspoons) and process this blood for later analysis. We intend to analyze your blood for markers of inflammation (c-reactive protein, tumor necrosis factor alpha, interleukin 6, interleukin 10, and monocyte chemoattractant protein 1), oxidative stress (F2 isoprostane, isofurans and malondialdehyde ) markers that tell us about the functioning of your blood vessels (endothelin-1 and nitrate/ nitrite) and markers that tell us how your kidneys are working (Indoxyl Sulfate (IS) and p-Cresyl Sulfate (pCS) . The PI, Dr. Headley or his Graduate Assistant, Dr. Kristyn Kirton ,or Dr. O’Neill a professor at the College, will take your blood or Tesia Pollock, a phlebotomist.
- Following taking the blood samples we will give you a beverage like Gatorade or fruit juice to drink.
- We will ask you to complete a series of questionnaires to assess quality of life (KDQOL scale), stress (Perceived Stress Scale), Anxiety (Beck Anxiety Inventory), Mood (Visual Analogue Scale), and food frequency.
- At the completion of this visit you will be paid $20

**Group assignment**

**Nutrition:** Following the completion of Visit 2 you will be assigned to one of **four** groups. Each group will be given a **powder to add to your foods** and beverages daily for sixteen weeks. This powder will either contain a corn starch-based fiber supplement that your body cannot digest (HiMaize 260) or regular corn starch. From week 2 onwards, your powder will contain 12 to 30 grams of carbohydrates, less than 1 g protein, and approximately 50 to 80 calories per day. Neither you nor most members of the research team will know which substance you are taking until the study is finished.

**Exercise:** Due to the COVID-19 pandemic we have excluded all of the exercise components of the study

**Visit 3:**

After 8 weeks of the study you will be asked return to the laboratory at Springfield College after an 8 hour fast. We will also ask you to log the foods that you eat for three days and bring that record along with you. We will assess the following:

- We will record your weight and body fat
- Blood pressure, and the stiffness of your blood vessels.
- We will take a blood sample to analyze it for the same blood markers as we did in visit 2.

At the completion of this visit you will be paid $ 20.

**Visit 4:**

Following the completion of the 16- week intervention you will return to the laboratory at Springfield College at approximately the same time of day as in your previous visits for your last visit which will involve some of the same measurements that were taken during Visit 2. Please see the list below:

- Prior to coming to the lab you will be asked to collect your stool sample along with your food logs as previously done.
- We will ask you to wear the monitor on your thigh for a seven-day period prior to coming for your final visit. The study coordinator will contact you regarding a time to have this device attached to your thigh.
- You will be expected to come to the lab after at least an 8 hour fast.
- We will once again measure your weight and body fat as we did at your first visit.
- We will ask you to complete a short computerized task to assess cognitive and emotional processing.
- After 10 minutes of supine rest, we will assess your blood pressure in your arm and the stiffness of your vessels.
- When the blood pressure measurements have been taken, we will take a blood sample (approximately 3 teaspoons) and process this blood for later analysis. We intend to analyze your blood for the same markers as previously detailed.
- Following taking the blood samples we will then ask you to complete the perceived stress questionnaire, the Beck Anxiety Inventory, the Visual Analog Scale, and the Food Frequency Questionnaire.

At the completion of this visit you will be paid $ 40

1. **The risks and the safeguards taken to minimize the risks are as follows:**

Most individuals who have used this fiber supplement have tolerated it very well. There have been no differences reported in digestive issues between those taking the fiber supplement and those taking the regular corn starch. To help your body adjust to your assigned supplement (fiber or regular corn starch), you will receive half the usual dose for the first week of the study. If you are experiencing any digestive issues, such as new problems with gas or loose stools, please tell the researchers immediately and you will be given appropriate instructions.

**IV. Benefits to you are as follows:**

You will be assessed on a number of tests including blood markers of inflammation and oxidative stress. You will also have your diet, psychological well-being, and cognitive functioning assessed. We will evaluate your blood pressure and the stiffness of your blood vessels. These evaluations will be done at no cost to you and at the completion of the study all of these results will be available to you or your physician if you so choose.

At the completion of the study, you will be given a free three-month membership at the YMCA of greater Springfield, Westfield or Scantic Valley in Wilbraham.

**You will be paid $100 for your involvement in this study. You will be paid this sum over the duration of the study using the following schedule; $20 at visits 1, 2 and 3 and the remaining $40 at the end of visit 4 when you have completed the entire study. Failure to return the monitor will result in you not receiving this final payment.**

V. **Confidentiality**

The information obtained about you will be kept in confidence, although you are free to release it to your own physician. The information will be used for statistical or scientific purposes without identifying you as an individual.

Any significant new findings will be provided to you during the course of the study. You are free to withdraw from this project at any time without penalty or loss of benefits to which you would otherwise be entitled. Should a physical injury occur, appropriate first aid will be provided, but no financial compensation will be given. Further information can be obtained from the Office of Academic Affairs at Springfield College concerning pertinent questions about the research and an explanation of your rights as a research subject. The Office of Academic Affairs serves as the official contact office in the event of research related injury to you (413-748-3959). The email address and telephone number of Sam Headley, the principal investigator ([sheadley@springfieldcollege.edu](mailto:sheadley@springfieldcollege.edu), 413-748-3340) is included in the event that you want to contact him directly for an explanation of any aspect of this study.

**Disclosure**

This study is being supported by an internal grant from Springfield College.

All research staff are being compensated for their work on this project.

**I CERTIFY THAT I HAVE READ AND FULLY UNDERSTAND THE ABOVE PROJECT. ALL MY QUESTIONS HAVE BEEN ANSWERED TO MY SATISFACTION BY THE RESEARCH STAFF. I WILLINGLY CONSENT TO PARTICIPATE.**

**______________________ ____________________________**

**Signature of Witness Signature of Subject**

**_____________________ ______________________**

**Date Date**

**I CERTIFY THAT I HAVE EXPLAINED FULLY TO THE ABOVE SUBJECT THE NATURE AND PURPOSE, THE POTENTIAL BENEFIT AND FORESEEABLE RISK OF THE INDICATED PROCEDURE OR DRUG. I HAVE ANSWERED ALL QUESTIONS ASKED BY THE SUBJECT.**

**________________________________________________ _________**

**Signature of the person administering the consent form Date**
