# Supplementary material for: The effects of 16-weeks of prebiotic supplementation and aerobic exercise training on inflammatory markers, oxidative stress, uremic toxins, and the microbiota in pre-dialysis kidney patients: a randomized controlled trial-protocol paper
Source: BMC Nephrol. 2020 Nov 26;21:517. doi: 10.1186/s12882-020-02177-x (PMC7689649; doi:10.1186/s12882-020-02177-x)
Supplement: Supplementary file 2 — Additional file 2. [file 12882_2020_2177_MOESM2_ESM.docx]

Springfield College

Department of Exercise Science and Athletic Training

**Subject** Symptom Check Documentation

Please respond to the following questions:

In the last 12-24 hours have you experienced any of the following symptoms:

1. Cough _____YES _____NO
2. Shortness of Breath _____YES _____NO
3. Fever _____YES _____NO
4. Chills _____YES _____NO
5. Muscle Pain _____YES _____NO
6. Sore Throat _____YES _____NO
7. New loss of taste or smell _____YES _____NO

Temperature: __________ (taken prior to entering facility)

Name/Signature of Subject ________________________________________Date: __________

Signature of Researcher ________________________________________Date: __________
